# Manufacturer’s CORBA Interface Testing Toolkit: Overview

**DOI:** 10.6028/jres.104.015

**Published:** 1999-04-01

**Authors:** David Flater

**Affiliations:** National Institute of Standards and Technology, Gaithersburg, MD 20899-0001

**Keywords:** code generation, CORBA, software, testing

## Abstract

The Manufacturer’s CORBA Interface Testing Toolkit (MCITT) is a software package that supports testing of CORBA components and interfaces. It simplifies the testing of complex distributed systems by producing “dummy components” from Interface Testing Language and Component Interaction Specifications and by automating some error-prone programming tasks. It also provides special commands to support conformance, performance, and stress testing.

## 1. Introduction

Some years ago, object-oriented programming languages were introduced to improve the maintainability and reusability of software systems. The object-oriented approach permits developers to map real-world entities to programming language constructs called *classes* that encapsulate the attributes and behaviors needed to model the real-world entity. Usage of object-oriented techniques is motivated by the belief that they result in software that is more understandable as well as more maintainable and reusable.

Object-oriented programming languages are complemented by an object-oriented mechanism for communication between programs, where the programs run independently and may run on different physical computer systems. The “owner” program for a given object creates the object and uses the program memory resources to store the state (data) of the object. When a program invokes an operation on an object that it owns (a “local” object), it is just like a function call; but when a program invokes an operation on an object that is owned by a different program (a “remote” object), the request and its response must be transmitted over the network. Distributed object architectures, such as the Common Object Request Broker Architecture (CORBA), [1] provide this network service so that invoking operations on remote objects is no harder for the programmer than invoking operations on local objects. This capability leads to the construction of “distributed object systems” in which the conceptual design of a distributed system is mapped onto a distributed object implementation. Because of their maintainability, performance, and standardization, distributed object architectures are being examined by many as a possible replacement for aging and inadequate software infrastructures.

The Common Object Request Broker Architecture (CORBA) supports the construction of distributed systems containing many components. These components can interact in complex ways, not necessarily conforming to a strict client-server model. This generality is necessary to enable many real-world systems to be built on a distributed architecture. However, it is also the reason why testing these systems is so difficult. Because each component can have complex dependencies on any number of other components, it is often impossible to test them in isolation.

The Manufacturer’s CORBA Interface Testing Toolkit (MCITT, pronounced “M-kit”) mitigates this problem by minimizing the amount of effort needed to produce simple emulations of servers, or *dummy components*, [2] that can be used to replace actual servers in a testing scenario. The person doing the testing only needs to specify the behaviors that are important for the specific scenario being examined, and MCITT will do the rest. CORBA boilerplate code, memory management, and stubs for unused operations are generated automatically.

Dummy components are useful for unit testing, where one component must be tested in isolation; for system testing and integration testing when some components are not available or not trusted; and for conformance testing, to provide a more controlled testing environment. MCITT provides additional services in support of conformance and performance testing with specialized Interface Testing Language (ITL) constructs: conformance test assertions, automatic inclusion of conformance test boilerplate, and timed loops for performance evaluation. Test assertions can even be derived automatically from Component Interaction Specifications (CISs).

MCITT was produced in the Manufacturing Engineering Laboratory of the National Institute of Standards and Technology (NIST) in support of the Advanced Process Control Framework Initiative (APCFI) [3] and the National Advanced Manufacturing Testbed Framework Project. [4] While not all possible features have been implemented, the toolkit is already quite useful in its current form, and sufficient groundwork for continued development and commercialization by private companies has been laid. A license statement detailing all of the relevant intellectual property issues is provided on the MCITT FTP site [5] and in the distribution. The toolkit is free for non-commercial use; easily surmountable restrictions on commercialization are explained in the license.

Despite its automation of various labor-intensive tasks, MCITT is not an “automatic testing tool.” To call it an “automatic testing tool” would imply that it generated test cases directly from a formal specification of the software, or that it used fault injection, [6] capture/playback, [7] or other potentially “context-free” techniques to conduct some kind of testing in the absence of a formal specification. Instead, MCITT relies on the skills and expertise of the person who designs the tests while reducing the amount of time that that person spends on menial tasks. MCITT can be used in the absence of a formal specification for the software under test, and its testing is not limited to what can be expressed in a formal language. Hand-written test code can easily be mingled with MCITT-generated code, should this become necessary. On the other hand, such things as pre-and post-conditions and assertions that appear in formal specifications also translate easily into MCITT’s specification languages.

## 2. Operation

There are two complementary ways of defining behaviors for dummy components: the procedural way, using Interface Testing Language (ITL), and the declarative way, using Component Interaction Specifications (CISs). Each way has its own features and limitations, but they are not mutually exclusive. Some behaviors of a server can be derived from CIS while others are specified in ITL.

**Fig. 1:**
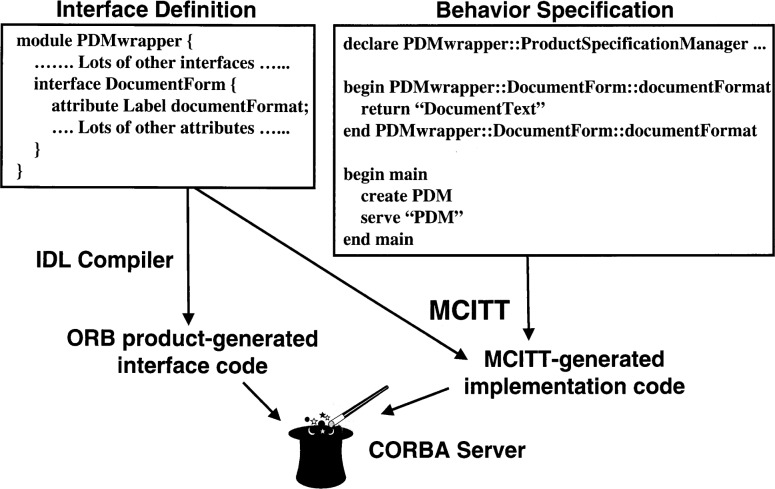
Building a server with MCITT.

Figure 1 illustrates the process by which a server is built using MCITT. The process is similar to that used to build a CORBA server from IDL (Interface Definition Language) and a C++ implementation, but in this case the C++ implementation is generated by MCITT from the Interface Testing Language and/or Component Interaction Specification files that you provide. The “big win” is that it is less labor-intensive and less error-prone to specify the behaviors needed for a testing scenario in ITL or CIS, letting MCITT generate all of the boilerplate code and stubbed-out operations, than it is to implement the entire scenario directly in C++. A “smaller win” is that equivalent servers can then be generated for different platforms (different CORBA products, different operating systems, and possibly different programming languages) with relative ease by selecting different *bindings* when MCITT is invoked. (Bindings will be explained in greater detail later in this article.)

### 2.1 Toolkit Contents

All of the previously described functionality pertains to **itlc**, the ITL compiler. This is only one of the tools in MCITT, which also contains **idlmkmf** (“IDL make makefiles”) and **TEd** (the NIST Test Editor).

**TEd** is a tool for noninteractive editing of test scripts and programs. It is used, for example, to automate the conversion of ITL files from a C++ binding to work with the Java binding. Written by Chris Schanzle for the NIST SQL Test Suite, [8] it provides much of the functionality of the standard Unix tool **sed**, [9] but it is more portable and has some operational differences that make it better suited for editing test cases. Because of its usefulness in this regard, it has been included in MCITT.

**Idlmkmf** generates makefile rules for all of the IDL files in the working (current) directory, plus a few useful variable definitions. Then it is only necessary to add rules specific to the clients and servers being built to produce a complete makefile for the CORBA application.

### 2.2 Interface Testing Language

Interface Testing Language (ITL) is a simplified procedural language for specifying and testing the behavior of CORBA clients and servers. The ITL compiler, **itlc**, uses a set of code template files called a *binding* to translate ITL into an implementation language. Different bindings can be created to absorb the differences between one platform and the next, thus achieving a higher degree of platform independence.

Currently, the MCITT distribution comes with bindings for Orbix 2 with the Sun C++ compiler (Solaris) and the Visual C++ compiler (Windows NT 4.0), and a partial binding for OrbixWeb 3.^1^ Bindings for other platforms are easily created by copying and modifying the existing bindings with a text editor. The platform changes are then automatically applied to all ITL compilations that select the new binding.

ITL provides the following services:

- Common operations that require messy blocks of code in C++ are condensed into single statements.
- Specialized support is provided for conformance testing, performance testing, and server emulation.
- Interfaces and operations that are not relevant to the scenario under test can be omitted completely.
- Memory management, TIE creation, and other “CORBA noise” are eliminated.
- Interfaces can be specified generically using their IDL names, or different names can be assigned to specific server object instances.

ITL features include run-time assertions, script-like behavior specifications, conformance testing commands, timed loops, and simple commands for creating and binding to CORBA objects.

While it would drastically lengthen this article to include a full explanation of the ITL language, a simple example would nonetheless be helpful. For a detailed explanation of the meaning of any of the ITL commands included in the following example, please consult the documentation provided in the MCITT distribution. [10]

Given the following IDL:


// Interface definition for “Hello,
world” server
interface Hello {
string says ();
};


A server can be implemented with this ITL:


// Behavior definition for “Hello,
world” server
declare Hello smiley “hello.hh”
“localhost” “hello”
begin smiley::says
return “Hello, world!”
end smiley::says
begin main
create smiley
serve “hello”
end main


This example does not demonstrate many of the useful features of ITL, but it does show how the details of a CORBA server main program—creating objects, creating TIEs for those objects, setting marker names and timeouts, invoking the CORBA main loop, catching exceptions—are condensed to the bare minimum.

### 2.3 Component Interaction Specifications

A Component Interaction Specification, or CIS, is a textual specification of an interaction scenario for an entire distributed system. A CIS can be used to generate servers that will execute the scenario that it describes.

Unlike an ITL file, which specifies the behavior for one component only, a CIS can describe all of the interactions between all of the components in the system. This unified CIS can then be referenced by minimal ITL files for all of the components, and the behaviors pertaining to each individual component will be distilled out of the CIS and generated by MCITT. (The ITL is still needed to declare identifiers and to specify what server objects should be created on startup.)

An interaction scenario consists of a tree of CORBA requests having specified inputs, outputs, and/or return values. The tree is rooted at the test client that initiates the entire chain of events. In order to capture the tree structure of the interactions in a flat ASCII script, an outline numbering convention similar to that of Unified Modeling Language Collaboration Diagrams [11] is used:


1 … first request by testing client on server A …
2 … second request by testing client on server A …
 2.1 … request by server A on server
B …
 2.2 … request by server A on server
C …
3 … third request by testing client …


**Fig. 2:**
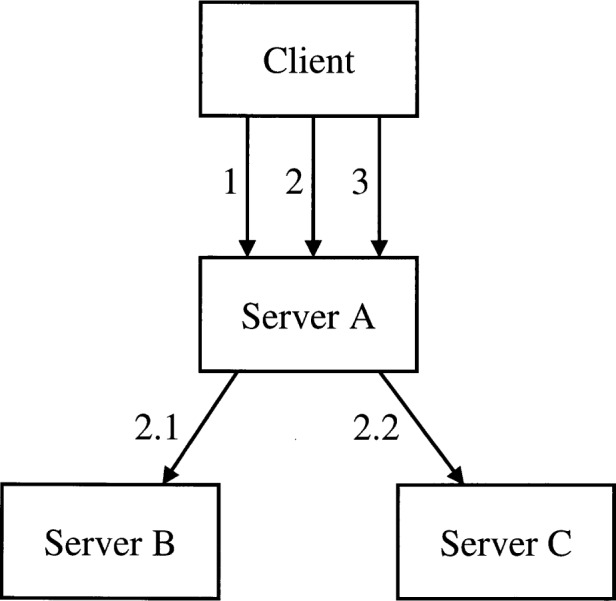
Illustration of Component Interaction Specification example.

The meaning of the above outline is that the requests on server B and server C are both made by server A before it responds to the second request made by the test client. That is, the implementation of the operation requested in the testing client’s second request makes requests on servers B and C before it returns a result to the testing client (see Fig. 2).

At this time, CIS processing is limited to generating the behaviors on the server side of the interactions. The requests must still be done manually in ITL or added to the generated source code using **TEd**. (This limitation may be removed in future development of MCITT; for more information, see the section on limitations that appears later in this article.)

MCITT’s CIS syntax is based on the Object Interaction Diagrams, or OIDs, used by NIST’s industrial partners in the Advanced Process Control (APC) Framework Initiative. The format originally used by APC to describe each request is like the following example:


1.3 install_component
Message from System Manager GUI to System Manager server
Format void install_component(System-Manager::ComponentName)
Actual Message:
{
“DataStore”
}


MCITT’s CIS makes the following cosmetic changes to the APC format:

- Remove redundant information;
- Replace informal component/object names with meaningful identifiers;
- Replace “Actual Message:” and “Actual Return Structure:” (not shown in this example) with simply In: and Out:

A CIS file consists of a sequence of messages having the following form:


1.2 interface::operation
In: { … argument values … }
Out: { return value, out values … }


The In: and Out: lists are both optional. The In: list specifies input values for all parameters whose attribute is **in** or **inout**; the Out: list specifies output values for the return and for all parameters whose attribute is **out** or **inout**. The return value, if any, is the first item in the Out: list.

The In: list will be used to construct the request on the client side (when implemented), and is used to perform assertion checking of the received values on the server side. Similarly, the Out: list is used to construct return values and out-values, and (when implemented) to perform assertion checking of the received values on the client side.

The syntax of the In: and Out: lists of CIS follows that of the “Actual Message:” and “Actual Return Structure:” lists of APC:

- String: “data”
- Sequence: #(value, value)
- Integer: 42
- Float: 42.6667
- Character: ‘d’
- Structure: {value, value}
- Union: [tag, value]
- Object (RepositoryID): SystemManager::Widget
- Boolean: true
- Octet: 0×20
- Any: {typecode, value} (not supported at this time) Sequences, structures, and unions may be nested to construct arbitrarily complex values. Here is how the previous example from APC would be rewritten in CIS format:


1.3 SystemManager::install_component
In: {“DataStore”}


In addition to generating the results specified in the CIS, servers generated from CIS will verify at run-time that their in-parameters all have the expected values. For example, the following request in a CIS:


1 ChipShooter::newParameter
In: {“rate”, 240.0, “placements per minute”}
Out: {Parameter}


will generate code that is equivalent to this ITL (again, please see Ref. [10], the ITL documentation, for a detailed explanation of commands):


begin ChipShooter :: newParameter
begin script
 call 1
  assert name == “rate”
  assert initval == 240.0 +− 1e-5
  assert units == “placements per minute”
  create newparm
  return newparm
 // Subsequent calls go here…
end script
end ChipShooter::newParameter


Finally, perhaps the biggest advantage of CIS is that complex, nested data objects can be constructed with relative ease by describing them in CIS syntax:


In: {# ({{{{32, 25, 0, 7}, {6, 90,
2002}}, “ID_RONETEU”, “50”, # ()},# ({
// Et cetera…


## 3. Capabilities

Note: Ref. [7] provides useful, simple definitions and additional discussion for most of the following varieties of software testing.

### 3.1 Unit Testing

Before a component is integrated into a distributed system, it should be tested in isolation to find and remove the obvious bugs. However, the developers of a component may be discouraged by the amount of scaffolding and throwaway code that is required to enable unit testing to take place. Any requests that a component would make on an external server must be stubbed out, or else a dummy server must be produced. MCITT reduces the effort of unit testing by partially automating the production of dummy servers.

### 3.2 Integration Testing

It is painful indeed to go through the effort of implementing and unit-testing multiple components only to find out in integration testing that ambiguities or inconsistencies in the interface specifications resulted in implementations that cannot work together. MCITT enables these problems to be found at an earlier stage by producing dummy components long before the real components are finished. The interface specifications can be “validated” using all dummy components before implementation of the real components ever begins. Design problems can then be corrected at a much less expensive stage.

### 3.3 System Testing

A piece of bad data may propagate through several components before a problem ever appears. Locating the fault in such cases can be difficult. A good strategy to narrow the possibilities is to replace one or more of the components with dummy components to see whether the problem goes away. A dummy component anywhere on the path of the bad data will break the chain and cause correct operation, so the fault is eventually located by walking backwards to the source.

Along the way, swapping dummy components for real components may detect hidden deviations from the specification, where a system is working only because all off the components using a given interface use the same *incorrect* interpretation, and are hence “bug-compatible” by accident. Incorrect but functional usage of an interface can propagate through a software project like a virus because developers will copy or re-use working code. Since the generation of testing scenarios for MCITT is isolated from the iterative programming and debugging task, they are more likely to resist “infection” and report assertion failures for incorrect usage. Then the code can be changed to match the documentation, or vice-versa.

### 3.4 Performance Testing

MCITT supports performance testing with a specialized ITL construct for timed loops (**begin timedloop, end timedloop**). With the exception of deducting a tare for the minimal overhead of the timing code itself, which is easily determined by timing an empty loop,^2^ all of the needed scaffolding is produced automatically. This feature was used in support of the APCFI to measure the CPU overhead incurred by CORBA (e.g., memory allocation, marshalling, and demarshalling). It was found that the complexity and nesting of data structures had a measurable impact on the CPU overhead, but the total CPU overhead in all cases was not a significant factor affecting the performance of the Advanced Process Control system overall.

### 3.5 Conformance Testing

The conformance testing methodology supported by MCITT is modeled on that of the NIST SQL Test Suite, with automation of some tasks that were previously done by hand. Each conformance test follows a pattern that culminates with the reporting of a test verdict. The verdicts are expected to be collected and summarized by an external reporting system, which is not included with the MCITT distribution. The code that MCITT generates for conformance tests can be customized to work with a particular test harness and test reporting system simply by modifying the binding.

A conformance test can report a verdict of PASS, FAIL, or NOGO. PASS and FAIL indicate that the test was successfully performed, with the results as reported. NOGO indicates that there was an operational problem that prevented the test from being performed—for example, the attempt to connect to the ORB (Object Request Broker) timed out. If there are extreme operational problems, a test may fail to report a verdict; this is handled the same as NOGO.

The best policy on NOGO and missing results depends on the nature of the testing being performed. In the SQL conformance testing program, the tester examined each NOGO or nonreporting test and either got it to run to completion or promoted the verdict to PASS. In different testing contexts it may be more appropriate to equate NOGO with FAIL.

ITL includes special-purpose commands for the following tasks related to conformance testing:

- Identify the conformance test (**begin test**);
- Verify pre- and post-conditions with verbose logging of expected values, actual values, and sub-test verdicts (**assert**);
- Explicitly set NOGO and/or FAIL indicators (**nogo**, **fail**);
- End the test and report a verdict (**end test**).

MCITT also automatically generates the boilerplate code to report a NOGO condition if an unexpected exception is thrown at the test client.

### 3.6 Stress Testing

The ITL command **fork** causes the test program to split itself into *N* identical concurrent processes and resume execution only after all copies have become approximately synchronized with one another. This is useful for conducting stress tests on servers to see how many concurrent requests they can handle, to determine how many active CORBA processes a host machine can handle before resource contention becomes a problem, and to determine the effect of request volume on server response time.

In a particular test, it was found that attempts to connect to a CORBA server began failing *en masse* under relatively mild stress conditions (e.g., with a 4 s inter-arrival time on connection attempts), and response times on successful requests became unacceptably long. Resource contention was therefore identified as a significant threat to system performance, and the volume of requests would need to be taken into consideration when configuring the systems used in production.

It is possible to conduct other kinds of stress tests simply by using MCITT-generated clients and servers to increase the population of components in a distributed system. For example, if a control system is designed to handle hundreds of subordinate components, but it is not feasible to provide hundreds of real components with which to test it, MCITT-generated simulations may be substituted. This might more properly be called a scalability test.

## 4. Limitations

Due to finite resources, some features of MCITT for which there was no immediate need were not finished. Those who ultimately commercialize and maintain MCITT are encouraged to relax or eliminate these limitations.

A full list of limitations is given in the documentation within the distribution. Some minor limitations that are difficult to explain in brief have been omitted here.

### 4.1 Memory Management

MCITT is designed as a testing tool, not as a means for implementing servers for production use. As such, the emphasis is on simplicity and correct operation, and not on long-term stability. The practical ramification of this is that some MCITT constructs will cause memory leakage in generated servers. While it should not have any impact on servers used for testing purposes, this leakage may become noticeable if MCITT generated servers are (improperly) put into production use.

### 4.2 Data Types

The Any data type is only minimally supported. IDL containing Anys can be processed by MCITT, and Any values created with inline code can be returned through MCITT-generated functions. However, generation of Anys from ITL or CIS without the use of inline code is not supported.

IDL arrays are not supported. Sequences can be used instead.

### 4.3 Fork

The ITL command **fork** is not supported in the NT or Java bindings.

### 4.4 Reporting

In assertions for complex types (sequences, structs, unions), the actual value of the run-time data item is not printed. However, the assertion is properly checked. (A full comparison of its value against the expected value is performed, and the result is printed.)

### 4.5 Java Binding

The Java binding is only stable for small, simple interfaces. The causes of the failures encountered with more complex interfaces are not yet understood.

Because of the aforementioned failures, work on the Java binding was abandoned with only simple data types, input parameters, and return values supported. Complex data types and parameters of mode *out* or *inout* are not supported or tested.

Some minor syntax changes are needed to change ITL files from C++ to Java. For example, embedded use of C++ pointer notation (−>,*) must be changed to Java syntax. Most of the actual work can be automated with **TEd** as is done in the demos provided with the distribution.

### 4.6 CIS Limitations

Generating requests from CIS is not supported. Only assertion-checking and response generation are done.

The assertion checking in generated servers will not always detect deviations from the CIS that consist of the correct messages happening in the wrong order.

There is no way to specify exceptions in a CIS.

## 5. Conclusion

Software testing today is still more art than science. While formal approaches may lend a feeling of rigor to software testing, it is unlikely that the results of software testing will ever be as reliable as the results of physical experiments, as we would hope. The reason is simply that the run-time environment contains too many hidden, unknown, and/or uncontrollable variables for it to serve as a laboratory. The industrial user of shrink-wrapped software components can neither predict nor control what faults they may introduce into the system, and few people are surprised any more when components just “blow up” for no known, reproducible reason. Often these disasters result from changes in the operating system and file system environment that are, on the face of things, completely irrelevant to the crashed program. In such cases, the obviously incomplete specifications are of little assistance, and it is only by years of experience with the entire operating environment that a skilled engineer may know where to look for the problem. It is hoped that MCITT will provide some practical assistance to the engineers who must prevent or diagnose such failures.

## Footnotes

1 Certain commercial equipment, instruments, or materials are identified in this paper to foster understanding. Such identification does not imply recommendation or endorsement by the National Institute of Standards and Technology, nor does it imply that the materials or equipment identified are necessarily the best available for the purpose.

2 The overhead in question is simply the reading of the system clock. The tare was found to be a mere 2 μs on one of our testing platforms, and too far below the resolution of the clock to be measured with any accuracy on another, so we generally disregard the tare.
